# Supplementary material for: Influence of renal function and daptomycin dose on clinical effectiveness and adverse events in Japanese pediatric patients: A multicenter retrospective observational study
Source: PLoS One. 2025 Jul 17;20(7):e0327993. doi: 10.1371/journal.pone.0327993 (PMC12270112; doi:10.1371/journal.pone.0327993)
Supplement: S1 Table — (DOCX) [file pone.0327993.s001.docx]

Supplemental Table 1. Recommended dose adjustments for daptomycin in pediatrics with renal impairment [22]

| Infection | Age, years | eGFR, mL/min/1.73 m^2^ | | | | |
| --- | --- | --- | --- | --- | --- | --- |
|  |  | ≥90 | 60–89 | 30–59 | 15–29 | <15 |
| cSSTI | 12–17 | 5 mg/kg q24h | No dosage adjustment need | | 5 mg/kg q48h | 5 mg/kg q48h |
|  | 7–11 | 7 mg/kg q24h |  |  | 7 mg/kg q48h | 7 mg/kg q48h |
|  | 2–6 | 9 mg/kg q24h |  |  | 9 mg/kg q48h | 7 mg/kg q48h |
|  | 1–2 | 10mg/kg q24h |  |  | 10 mg/kg q48h | 6 mg/kg q48h |
| Bacteremia | 12–17 | 7 mg/kg q24h | No dosage adjustment need | | 7 mg/kg q48h | 7 mg/kg q48h |
|  | 7–11 | 9 mg/kg q24h |  |  | 9 mg/kg q48h | 9 mg/kg q48h |
|  | 1–6 | 12 mg/kg q24h |  |  | 12 mg/kg q48h | 8 mg/kg q48h |

cSSTI, complicated skin and skin structure infection; eGFR, estimated glomerular filtration rate
