# Supplementary material for: Influence of renal function and daptomycin dose on clinical effectiveness and adverse events in Japanese pediatric patients: A multicenter retrospective observational study
Source: PLoS One. 2025 Jul 17;20(7):e0327993. doi: 10.1371/journal.pone.0327993 (PMC12270112; doi:10.1371/journal.pone.0327993)
Supplement: S2 Table — (DOCX) [file pone.0327993.s002.docx]

Supplemental Table 2. Infectious diseases of excluded patients except for bacteremia or cSSTI (n = 17)

|  | n |
| --- | --- |
| Fever | 4 |
| Vascular infection | 2 |
| Otitis externa | 2 |
| Arthritis | 3 |
| Urinary tract infection | 2 |
| Marrow meningitis, enteritis, bone marrow peritonitis | 1 |
| Pin-site infection | 1 |
| Fever, suspected enteritis | 1 |
| Fever, cholangitis | 1 |

cSSTI, complicated skin and skin structure infection
