# Supplementary material for: Influence of renal function and daptomycin dose on clinical effectiveness and adverse events in Japanese pediatric patients: A multicenter retrospective observational study
Source: PLoS One. 2025 Jul 17;20(7):e0327993. doi: 10.1371/journal.pone.0327993 (PMC12270112; doi:10.1371/journal.pone.0327993)
Supplement: S3 Table — (DOCX) [file pone.0327993.s003.docx]

Supplemental Table 3. Bacteria and MIC values for patients with positive blood cultures (n = 21)

| Case | Bacteria | MIC | Type of Infection | Dose |
| --- | --- | --- | --- | --- |
| 1 | *Leuconostoc lactis* | – | Bacteremia | Underdose |
| 2 | MRSA | 0.25 | Bacteremia | Adequate dose |
| 3 | MRSA | 0.25 | cSSTI | Overdose |
| 4 | MRSA | 0.5 | Bacteremia | Overdose |
| 5 | MRSA | <1.0 | Bacteremia | Adequate dose |
| 6 | MRSE | 0.5 | Bacteremia | Adequate dose |
| 7 | MRSE | 1.0 | Bacteremia | Adequate dose |
| 8 | MRSE | – | Bacteremia | Adequate dose |
| 9 | MRSE | 0.5 | Bacteremia | Adequate dose |
| 10 | MSSA | <0.25 | Bacteremia | Adequate dose |
| 11 | *Staphylococcus epidermidis* | <0.25 | Bacteremia | Underdose |
| 12 | *Staphylococcus epidermidis*, MRCNS | <0.5 | Bacteremia | Adequate dose |
| 13 | *Staphylococcus epidermidis*, MRCNS | 2.0 | Bacteremia | Adequate dose |
| 14 | *Staphylococcus epidermidis*, MRCNS | <0.5 | Bacteremia | Adequate dose |
| 15 | *Staphylococcus epidermidis*, MRCNS, *Streptococcus oralis* | <0.5 | Bacteremia | Adequate dose |
| 16 | *Staphylococcus haemolyticus*, MRCNS | <0.5 | Bacteremia | Adequate dose |
| 17 | *Staphylococcus hominis*, MRCNS | <0.5 | Bacteremia | Adequate dose |
| 18 | *Staphylococcus haemolyticus* | 0.5 | Bacteremia | Adequate dose |
| 19 | *Staphylococcus epidermidis* | 1.0 | Bacteremia | Adequate dose |
| 20 | *Staphylococcus epidermidis* | 0.5 | Bacteremia | Adequate dose |
| 21 | *Staphylococcus haemolyticus* | 0.5 | cSSTI | Adequate dose |

cSSTI, complicated skin and skin structure infection; F, female; M, male; MIC, minimum inhibitory concentrations; MRCNS, methicillin-resistant *coagulase-negative Staphylococci*; MRSA, methicillin-resistant *Staphylococcus aureus*; MRSE, methicillin-resistant *Staphylococcus epidermidis*; MSSA, methicillin-susceptible *Staphylococcus aureus*
