# Supplementary material for: Influence of renal function and daptomycin dose on clinical effectiveness and adverse events in Japanese pediatric patients: A multicenter retrospective observational study
Source: PLoS One. 2025 Jul 17;20(7):e0327993. doi: 10.1371/journal.pone.0327993 (PMC12270112; doi:10.1371/journal.pone.0327993)
Supplement: S4 Table — (DOCX) [file pone.0327993.s004.docx]

Supplemental Table 4. Age-stratified clinical characteristics of patients

|  | 1–<2 years (n = 2) | 2–6 years (n = 8) | 7–11 years (n = 4) | 12–17 years (n = 40) |
| --- | --- | --- | --- | --- |
| Age, years | 1.0 [1.0–1.0] | 2.5 [2.0–4.0] | 9.5 [7.3–11.0] | 17.0 [12.0–18.1] |
| Male, n (%) | 1 (50) | 4 (50) | 3 (75) | 25 (63) |
| Weight, kg | 13.2 [11.3–15.0] | 15.6 [10.6–19.1] | 32.5 [22.9–43.9] | 50.8 [19.3–105.0] |
| Treatment duration, day | 16.0 [6.0–26.0] | 23.5 [3.0–94.0] | 28.0 [21.0–35.0] | 14.0 [3.0–106.0] |
| Haemodialysis, n (%) | 1 (50) | 0 (0) | 0 (0) | 0 (0) |
| Continuous haemodiafiltration, n (%) | 0 (0) | 1 (13) | 1 (25) | 2 (5) |
| Renal function in patients without dialysis therapy, n (%) | 1 (50) | 7 (88) | 3 (75) | 38 (95) |
| eGFR ≥90, mL/min/1.73 m^2^, n (%) | 0 (0) | 5 (63) | 3 (75) | 34 (85) |
| eGFR 60–89, mL/min/1.73 m^2^, n (%) | 1 (50) | 0 (0) | 0 (0) | 2 (5) |
| eGFR 30–59, mL/min/1.73 m^2^, n (%) | 0 (0) | 2 (25) | 0 (0) | 1 (3) |
| eGFR 15–29, mL/min/1.73 m^2^, n (%) | 0 (0) | 0 (0) | 0 (0) | 0 (0) |
| eGFR <15, mL/min/1.73 m^2^, n (%) | 0 (0) | 0 (0) | 0 (0) | 1 (3) |
| Alb, g/dL | 3.6 [3.4–3.7] | 2.9 [1.3–4.4] | 3.5 [2.4–4.6] | 3.5 [1.9–4.7] |
| BUN, mg/dL | 38.6 [12.8–64.4] | 11.5 [3.5–70.3] | 9.7 [7.0–35.2] | 12.5 [5.0–78.0] |
| Scr, mg/dL | 0.6 [0.4–0.7] | 0.2 [0.1–0.9] | 0.4 [0.3–0.7] | 0.6 [0.1–7.1] |
| AST, U/L | 33.0 [33.0–33.0] | 30.0 [8.0–124.0] | 31.0 [19.0–124.0] | 21.0 [8.0–173.0] |
| ALT, U/L | 15.0 [15.0–15.0] | 15.5 [7.0–167.0] | 20.0 [6.0–32.0] | 26.0 [8.0–184.0] |
| Hb, g/dL | 8.8 [8.7–8.8] | 9.5 [7.1–12.5] | 10.6 [9.8–13.4] | 9.6 [5.9–14.6] |
| CRP, mg/dL | 5.0 [1.0–9.1] | 3.5 [0.1–12.7] | 4.3 [0.9–8.0] | 1.2 [0.0–27.1] |
| Eosinophil count, /μL | 0.0 [0.0–0.0] | 1.0 [0.0–396.0] | 107.0 [107.0–107.0] | 12.0 [0.0–1120.0] |
| CPK, U/L | 45.0 [20.0–70.0] | 22.0 [6.0–86.0] | 50.0 [4.0–210.0] | 0.0 [0.0–3324.0] |
| cSSTI, n (%) | 0 (0) | 7 (88) | 3 (75) | 10 (25) |
| Bacteremia, n (%) | 2 (100) | 1 (13) | 1 (25) | 30 (75) |
| **Daptomycin dose** |  |  |  |  |
| Underdose, n (%) | 0 (0) | 3 (38) | 1 (25) | 0 (0) |
| Adequate dose, n (%) | 2 (100) | 4 (50) | 2 (50) | 32 (80) |
| Overdose, n (%) | 0 (0) | 1 (13) | 1 (25) | 8 (20) |
| **Concomitant medications** |  | | | |
| Statin, n (%) | 0 (0) | 0 (0) | 0 (0) | 0 (0) |
| Fibrate, n (%) | 0 (0) | 0 (0) | 0 (0) | 3 (8) |
| SSRI, n (%) | 0 (0) | 0 (0) | 0 (0) | 0 (0) |
| *β*-blocker, n (%) | 0 (0) | 1 (13) | 0 (0) | 1 (3) |
| Antipsychotics, n (%) | 1 (50) | 0 (0) | 0 (0) | 2 (5) |
| Colchicine, n (%) | 0 (0) | 0 (0) | 0 (0) | 0 (0) |
| Steroids, n (%) | 0 (0) | 4 (50) | 0 (0) | 8 (20) |
| Amiodarone, n (%) | 0 (0) | 0 (0) | 0 (0) | 0 (0) |
| Cyclosporine, n (%) | 0 (0) | 0 (0) | 0 (0) | 3 (8) |
| Propofol, n (%) | 0 (0) | 0 (0) | 0 (0) | 3 (8) |
| Antihistamine, n (%) | 1 (50) | 2 (25) | 1 (25) | 7 (18) |
| **Clinical effectiveness** |  |  |  |  |
| Cure, n (%) | 1 (50) | 4 (50) | 2 (50) | 16 (40) |
| Improvement, n (%) | 1 (50) | 1 (13) | 1 (25) | 13 (33) |
| Failure, n (%) | 0 (0) | 2 (25) | 1 (25) | 1 (3) |
| Non-evaluable, n (%) | 0 (0) | 1 (13) | 0 (0) | 10 (25) |
| Death, n (%) | 0 (0) | 2 (25) | 1 (25) | 1 (3) |
| **Microbiological effectiveness** |  |  |  |  |
| Initially negative, n (%) | 0 (0) | 4 (50) | 3 (75) | 23 (58) |
| Disappear, n (%) | 1 (50) | 2 (25) | 1 (25) | 12 (30) |
| Non-evaluable, n (%) | 0 (0) | 0 (0) | 0 (0) | 5 (13) |
| Microbiological failure, n (%) | 1 (50) | 2 (38) | 0 (0) | 0 (0) |

Alb, serum albumin; ALT, alanine aminotransferase; AST, aspartate transaminase; BUN, blood urea nitrogen; CPK, creatine phosphokinase; CRP, C–reactive protein; cSSTI, complicated skin and skin structure infection; eGFR, estimated glomerular filtration rate; Hb, haemoglobin; Scr, serum creatinine; SSRI, selective serotonin reuptake inhibitor.
