# Supplementary material for: Influence of renal function and daptomycin dose on clinical effectiveness and adverse events in Japanese pediatric patients: A multicenter retrospective observational study
Source: PLoS One. 2025 Jul 17;20(7):e0327993. doi: 10.1371/journal.pone.0327993 (PMC12270112; doi:10.1371/journal.pone.0327993)
Supplement: S5 Table — (DOCX) [file pone.0327993.s005.docx]

Supplemental Table 5. Clinical characteristics of registered patients with and without daptomycin-related adverse events

|  | Daptomycin-related adverse events | | *P*–value |
| --- | --- | --- | --- |
|  | Yes (n = 7) | No (n = 47) |  |
| Age, years | 16.0 [1.0–18.1] | 16.0 [2.0–18.0] | 0.589 |
| 1–<2 years, n (%) | 2 (29) | 0 (0) | 0.004 |
| 2–6 years, n (%) | 0 (0) | 8 (17) |  |
| 7–11 years, n (%) | 0 (0) | 4 (9) |  |
| 12–17 years, n (%) | 5 (71) | 35 (74) |  |
| Male, n (%) | 5 (71) | 28 (60) | 0.693 |
| Weight, kg | 42.9 [11.3–83.0] | 44.8 [10.6–105.0] | 0.495 |
| Treatment duration, day | 9.0 [3.0–106.0] | 16.0 [3.0–94.0] | 0.303 |
| Haemodialysis, n (%) | 1 (14) | 0 (0) | 0.029 |
| Continuous haemodiafiltration, n (%) | 0 (0) | 4 (9) | 0.670 |
| Renal function in patients without dialysis therapy, n (%) | 6 (86) | 43 (98) | – |
| eGFR ≥90, mL/min/1.73 m^2^, n (%) | 5 (83) | 37 (84) | 0.110 |
| eGFR 60–89, mL/min/1.73 m^2^, n (%) | 1 (17) | 2 (5) |  |
| eGFR 30–59, mL/min/1.73 m^2^, n (%) | 0 (0) | 3 (7) |  |
| eGFR 15–29, mL/min/1.73 m^2^, n (%) | 0 (0) | 0 (0) |  |
| eGFR <15, mL/min/1.73 m^2^, n (%) | 0 (0) | 1 (2) |  |
| Alb, g/dL | 3.7 [3.4–4.2] | 3.5 [1.3–4.7] | 0.049 |
| BUN, mg/dL | 13.5 [10.0–64.4] | 12.0 [3.5–78.0] | 0.279 |
| Scr, mg/dL | 0.7 [0.4–0.8] | 0.5 [0.1–7.1] | 0.169 |
| AST, U/L | 21.5 [12.0–51.0] | 23.0 [8.0–173.0] | 0.529 |
| ALT, U/L | 26.0 [10.0–112.0] | 24.0 [6.0–184.0] | 0.977 |
| Hb, g/dL | 9.2 [8.6–13.3] | 9.7 [5.9–14.6] | 0.898 |
| CRP, mg/dL | 1.3 [0.1–12.8] | 2.1 [0.0–27.1] | 0.904 |
| Eosinophil count, /μL | 0.0 [0.0–580.0] | 16.0 [0.0–1120.0] | 0.520 |
| CPK, U/L | 8.0 [0.0–70.0] | 11.0 [0.0–3324.0] | 0.583 |
| cSSTI, n (%) | 1 (14) | 13 (28) | 0.696 |
| Bacteremia, n (%) | 6 (86) | 34 (72) |  |
| **Daptomycin dose** |  |  | 0.714 |
| Underdose, n (%) | 0 (0) | 4 (9) |  |
| Adequate dose, n (%) | 5 (71) | 35 (74) |  |
| Overdose, n (%) | 2 (29) | 8 (17) |  |
| **Concomitant medications** | | | |
| Statin, n (%) | 0 (0) | 0 (0) | 1.000 |
| Fibrate, n (%) | 0 (0) | 3 (6) | 0.730 |
| SSRI, n (%) | 0 (0) | 0 (0) | 1.000 |
| *β*-blocker, n (%) | 0 (0) | 2 (4) | 0.793 |
| Antipsychotics, n (%) | 0 (0) | 2 (4) | 0.793 |
| Colchicine, n (%) | 0 (0) | 0 (0) | 1.000 |
| Steroids, n (%) | 2 (29) | 10 (21) | 0.844 |
| Amiodarone, n (%) | 0 (0) | 0 (0) | 1.000 |
| Cyclosporine, n (%) | 0 (0) | 3 (6) | 0.730 |
| Propofol, n (%) | 0 (0) | 3 (6) | 0.730 |
| Antihistamine, n (%) | 1 (14) | 10 (21) | 0.846 |
| **Clinical effectiveness** |  |  |  |
| Cure, n (%) | 3 (43) | 20 (43) | 0.928 |
| Improvement, n (%) | 3 (43) | 13 (28) | 0.659 |
| Failure, n (%) | 0 (0) | 4 (9) | 0.670 |
| Non-evaluable, n (%) | 1 (14) | 10 (21) | 0.846 |
| Death, n (%) | 0 (0) | 4 (9) | 0.670 |
| **Microbiological effectiveness** |  |  |  |
| Initially negative, n (%) | 3 (43) | 27 (57) | 0.687 |
| Disappear, n (%) | 4 (57) | 12 (26) | 0.177 |
| Non-evaluable, n (%) | 0 (0) | 5 (11) | 0.612 |
| Microbiological failure, n (%) | 0 (0) | 3 (6) | 1.000 |

Alb, serum albumin; ALT, alanine aminotransferase; AST, aspartate transaminase; BUN, blood urea nitrogen; CPK, creatine phosphokinase; CRP, C–reactive protein; cSSTI, complicated skin and skin structure infection; eGFR, estimated glomerular filtration rate; Hb, haemoglobin; Scr, serum creatinine; SSRI, selective serotonin reuptake inhibitor.
