# Supplementary material for: Influence of renal function and daptomycin dose on clinical effectiveness and adverse events in Japanese pediatric patients: A multicenter retrospective observational study
Source: PLoS One. 2025 Jul 17;20(7):e0327993. doi: 10.1371/journal.pone.0327993 (PMC12270112; doi:10.1371/journal.pone.0327993)
Supplement: Attachment — Submitted filename: PONE-D-24-52543_Comments .docx [file pone.0327993.s006.docx]

Comments to Authors

The present article “Influence of renal function and daptomycin dose on clinical effectiveness and adverse events in Japanese pediatric patients: A multicenter retrospective observational study” involves study aimed to evaluate the clinical effectiveness and adverse events of daptomycin in pediatric patients, considering factors like dosage and renal function. A retrospective review of medical records from patients treated with daptomycin between 2011 and 2022 was conducted. Continuous monitoring is necessary, even for patients receiving the approved dose, to minimize adverse events. Adequate dosing was found to improve clinical outcomes, emphasizing the importance of proper dosage and renal function consideration in pediatric patients.

The article needs revision with the below concerns before further consideration.

Main concerns:

1. There are other similar studies available for example “Clinical Effectiveness, Safety Profile, and Pharmacokinetics of Daptomycin in Pediatric Patients: A Systematic Review”. What is the significance of this study considering the retrospective approach with 54 patients? Authors could have gone through different patient databases to include a larger sample size to obtain better conclusions.
2. Authors need to provide the reference from which they have decided the underdose, adequate and overdose. In the abstract the underdose value is mentioned less than 1mg/kg, however 6mg/kg is referred as underdose under clinical effectiveness on page 11.
3. How authors found this antibiotic successful in 98% as there is death of 7% and adverse events in 13% patients?
4. The manuscript is not very well organized, and several information were repeated like Fig 1 and Fig 2 legends are the same as mentioned in the manuscript text. Even the figure 2, the total is more than 100%.
5. The result of clinical effectiveness on page 11, is not representing any figure or table. Authors need to put either figure, table or supplemental file where these information are available.
6. Authors need to clarify the rationale of considering the adverse events and microbiological failure. They should discuss more about microbiological failure.
7. Authors need to provide the different infectious agents/bacterial species affected patient specific as additional column in a table. Ideally, the doses of daptomycin may have decided based on the MIC/MBC values depending on the bacterial species. However, it seems there is a specific dose decided to treat the patients.
8. As, this antibiotic is primarily recommended to be used as an alternative to the patients with antibiotic resistance (MRSA, MRSE etc), the results should discuss specifically the treatment outcomes of those cases.
