# Supplementary material for: Influence of renal function and daptomycin dose on clinical effectiveness and adverse events in Japanese pediatric patients: A multicenter retrospective observational study
Source: PLoS One. 2025 Jul 17;20(7):e0327993. doi: 10.1371/journal.pone.0327993 (PMC12270112; doi:10.1371/journal.pone.0327993)
Supplement: Attachment — Submitted filename: PONE-D-24-52543_Comments.docx [file pone.0327993.s008.docx]

Comments to Authors

The present article “Influence of renal function and daptomycin dose on clinical effectiveness and adverse events in Japanese pediatric patients: A multicenter retrospective observational study” involves study aimed to evaluate the clinical effectiveness and adverse events of daptomycin in pediatric patients, considering factors like dosage and renal function. A retrospective review of medical records from patients treated with daptomycin between 2011 and 2022 was conducted. Continuous monitoring is necessary, even for patients receiving the approved dose, to minimize adverse events. Adequate dosing was found to improve clinical outcomes, emphasizing the importance of proper dosage and renal function consideration in pediatric patients.

The article needs revision with the below concerns before further consideration.

Thank you for giving us the opportunity to strengthen our manuscript following your valuable comments and queries. We have incorporated your feedback and hope that our revisions persuade you to accept our manuscript.

Reviewer #1:

Main concerns:

1. There are other similar studies available for example “Clinical Effectiveness, Safety Profile, and Pharmacokinetics of Daptomycin in Pediatric Patients: A Systematic Review”. What is the significance of this study considering the retrospective approach with 54 patients? Authors could have gone through different patient databases to include a larger sample size to obtain better conclusions.

Response: Thank you for your comment. The proposed dosage of daptomycin for pediatric patients is based on age; however, dosage adjustments for those with renal impairment have not been well-established, as these patients were not included in clinical trials or previous studies. We have investigated the associations between clinical outcomes and risk factors, including daptomycin dosage and the presence of renal function. The approved doses were defined according to the daptomycin package insert and the presence of renal impairment (Front Pharmacol. 2022;13: 838599). Patients were classified based on the daptomycin dose (underdose: >1 mg/kg less than the approved dose; adequate dose: approved dose ±1 mg/kg; overdose: >1 mg/kg more than the approved dose). Our findings suggest that daptomycin is most effective when administered at the approved dose, as outlined in the package insert. Furthermore, we previously investigated the association between age and onset of daptomycin-induced adverse events using the U.S. food and drug administration adverse event reporting system (FAERS) [J Infect Chemother. 2025;31(1):102501.]. The FAERS database does not include clinical laboratory data, such as serum creatinine levels; thus, we could not evaluate renal functions as risk factors for daptomycin-related adverse events. Moreover, although daptomycin dosage was stratified by age and sources of infection, 5,084 reports lacked information on infection sources. Consequently, we were unable to assess the impact of daptomycin dosage. In this study, we addressed the lack of information such as data on patients with renal dysfunction. By conducting a multicenter collaborative study, we believe that we were able to not only increase the sample size, as pointed out, but also exclude site-specific data. Moving forward, it will be necessary to conduct further studies with an even larger sample size. We have added a sentence to this regard as follows: “The proposed dosage of daptomycin for pediatric patients is based on the age; however, dosage adjustments for those with renal impairment have not been well-established, because such patients were not included in clinical trials or previous studies. Furthermore, although we previously investigated the association between age and the onset of daptomycin-induced adverse events using the U.S. food and drug administration adverse event reporting system (FAERS) [J Infect Chemother. 2025;31(1):102501.], the FAERS database does not include clinical laboratory data, such as serum creatinine levels; thus, we could not evaluate renal functions as a risk factor for daptomycin-related adverse events. Additionally, while daptomycin dosage was stratified by age and sources of infection, 5,084 reports lacked information on infection sources. Consequently, we were unable to assess the impact of daptomycin dosage.” (Page 5, line 90-99)

“By conducting a multicenter collaborative study, we believe that we were able to not only increase the sample size but also exclude site-specific data.” (Page 19, line 278-279)

2. Authors need to provide the reference from which they have decided the underdose, adequate and overdose. In the abstract the underdose value is mentioned less than 1mg/kg, however 6 mg/kg is referred as underdose under clinical effectiveness on page 11.

Response: According to the U.S. FDA-approved labeling, the use of daptomycin at the recommended dose (approved dose), below the recommended dose (underdose), and above the recommended dose (overdose) had been documented. Although not specific to daptomycin, the definition was based on the definitions used for other medication (J Am Coll Cardiol. 2016;68(24):2597-2604.). Since daptomycin was administered based on body weight, we considered a 1 kg range and classified patients based on the daptomycin dose as follows: underdose (>1 mg/kg less than the approved dose), adequate dose (approved dose ±1 mg/kg), and overdose (>1 mg/kg more than the approved dose). We excluded cases where the evaluation based on renal function did not align with the evaluation based on body weight as shown in the page 9, line 188 to page 10, line 189. For pediatric patients aged 1-6 years with bacteremia and an eGFR >30 mL/min/1.73 m^2^, approved daptomycin dose is 12 mg/kg. Therefore, 6 mg/kg was categorized as underdose. We have added the reference and revised the sentence as follow: “Patients were classified based on the daptomycin dose according to the U.S. FDA-approved labeling (underdose, >1 mg/kg less than the approved dose; adequate dose, approved dose ±1 mg/kg; and overdose, >1 mg/kg more than the approved dose). Since daptomycin was administered based on body weight, we took into account a 1 kg range” (Page 7, line 123-127).

We revised the sentence “underdose (>1 mg/kg less than the approved dose)” and “overdose (>1 mg/kg more than the approved dose)” (Page 3, line 48-49)

Supplemental Table 1 Recommended dose adjustments for daptomycin in pediatrics with renal impairment

| Infection | Age, years | eGFR, mL/min/1.73 m^2^ | | | | |
| --- | --- | --- | --- | --- | --- | --- |
|  |  | ≥90 | 60–89 | 30–59 | 15–29 | <15 |
| cSSSI | 12–17 | 5 mg/kg q24h | No dosage adjustment need | | 5 mg/kg q48h | 5 mg/kg q48h |
|  | 7–11 | 7 mg/kg q24h |  |  | 7 mg/kg q48h | 7 mg/kg q48h |
|  | 2–6 | 9 mg/kg q24h |  |  | 9 mg/kg q48h | 7 mg/kg q48h |
|  | 1–2 | 10mg/kg q24h |  |  | 10 mg/kg q48h | 6 mg/kg q48h |
| Bacteremia | 12–17 | 7 mg/kg q24h | No dosage adjustment need | | 7 mg/kg q48h | 7 mg/kg q48h |
|  | 7–11 | 9 mg/kg q24h |  |  | 9 mg/kg q48h | 9 mg/kg q48h |
|  | 1–6 | 12 mg/kg q24h |  |  | 12 mg/kg q48h | 8 mg/kg q48h |

3. How authors found this antibiotic successful in 98% as there is death of 7% and adverse events in 13% patients?

Response: Clinical effectiveness was determined by physicians at the end of daptomycin therapy and was categorized as follows: cure, improvement, failure, and non-evaluable. The clinical success rate was defined as the sum of cases categorized as “cure” and “improvement” at the end of therapy. Three of the four patients categorized as “failure” were initially classified as non-evaluable due to missing information. However, upon further review, the cases involving death were reclassified as failure. Based on this approach, we revised the antibiotic success rate to 91%. When evaluating the efficacy of treatment “cure” or “improvement,” the primary focus is on the improvement or resolution of the patient's condition. Therefore, if an antibiotic successfully treats the infection-even with some side effects, it may still be considered effective, with outcomes like “cure” or “improvement.”

In summary, even in the presence of adverse events, if the antibiotic effectively treats the infection and leads to positive clinical results, it can still be classified as “cure” or “improvement.” A previous study also classified cases with adverse events or death as “cure,” “improvement,” or “non-evaluable” (Syriopoulou V, et al. Pediatr Infect Dis J. 2016;35:511-6.) (Page 3, line 51, Page 11, line 223, and Page 19, line 281)

4. The manuscript is not very well organized, and several information were repeated like Fig 1 and Fig 2 legends are the same as mentioned in the manuscript text. Even the figure 2, the total is more than 100%.

Response: Thank you for your comments. We have deleted the duplicated legends of Table1-2. We have revised as follows:

Figure 2

5. The result of clinical effectiveness on page 11, is not representing any figure or table. Authors need to put either figure, table or supplemental file where these information are available.

Response: Thank you for your suggestion. Excluding 10 patients for whom clinical effectiveness was not evaluable, the clinical success rate reached 91%, with 24 (55%) classified as cured and 16 (36%) showing improvement. As per your comment, we have added the clinical effectiveness as Figure 3. We revised the sentence as follows; “Overall clinical success was achieved in 40 patients. Excluding 10 patients for whom clinical effectiveness was not evaluable, the clinical success rate reached 91%, with 24 (55%) classified as cured and 16 (36%) showing improvement (Fig 3). Three cases of treatment failure involved patients who received an underdose of daptomycin for bacteremia.” (Page 11, line 222-225)

6. Authors need to clarify the rationale of considering the adverse events and microbiological failure. They should discuss more about microbiological failure.

Response: Previous report in adult patients have revealed that C_min_ (breakpoint of 24.3 mg/L) is most significantly associated with CPK elevation. Additionally, factors such as total body weight >111 kg, prior or concomitant therapy with a *β*-hydroxy-*β*-methylglutaryl–coenzyme A reductase inhibitor, and renal dysfunction have been identified as risk factors for CPK elevation, with more frequent monitoring recommended (Eur J Appl Physiol Occup Physiol. 1983;52:75–79.). However, a previous report in Japanese pediatric patients showed no apparent relationship between CPK elevations and daptomycin exposures (J Infect Chemother. 2023;29(6):592–598.). S4 Table presents the clinical characteristics of patients with and without daptomycin-related adverse events. There was no significant relationship between adverse events and patient characteristics, including weight, treatment duration, renal function, and daptomycin dose.

We have also revised the results as follows:

“Previous reports in adult patients have revealed that C_min_ ≥24.3 mg/L is most significantly associated with CPK elevation. Additionally, factors such as total body weight >111 kg, prior or concomitant therapy with a *β*-hydroxy-*β*-methylglutaryl–coenzyme A reductase inhibitor, and renal impairment have been identified as risk factors for CPK elevation (Eur J Appl Physiol Occup Physiol 1983;52:75–79.). However, in Japanese pediatric patients, no apparent relationship was observed between CPK elevations and daptomycin exposure (J Infect Chemother. 2023;29(6):592–598.). S4 Table presents the clinical characteristics of patients with and without daptomycin-related adverse events. No significant relationship was found between adverse events and patient characteristics, including body weight and renal function” (Page 20, line 320 to Page 21, line 328).

The bactericidal effect of daptomycin is generally associated with an AUC/MIC ≥200 [Expert Rev Clin Pharmacol. 2016;9:961–979], and greater therapeutic efficacy when the AUC/MIC ratio >666 [Intensive Care Med. 2020;46:1127–1153. Expert Rev Clin Pharmacol. 2016;9:961–979. Ther Drug Monit. 2018;40:589–595.] and a C_max_/MIC ratio between 12–94 is considered optimal for achieving bacteriostatic effects [Expert Rev Clin Pharmacol. 2016;9:961–979.] and minimizing bacterial resistance [Infect Chemother. 2013;19:732–739.]. Daptomycin has proven effective when administered at the approved dosage or higher. Some experts recommend higher dosages of daptomycin for MRSA bacteremia treatment [Clin Infect Dis. 2011;52:285–292.], and ineffectiveness in some patients may be attributed to suboptimal dosing [J Antimicrob Chemother. 2018;73(9):2277–2283.].

We have also revised the result as follows:

“Microbiological effectiveness was categorized as initially negative (n=30), disappeared (n=16), non-evaluable (n=5), or microbiological failure (n=3) (Table 1). Microbiological failure was significantly higher in patients receiving an underdose of daptomycin (*P* < 0.001)” (Page 12, line 226–228).

“The bactericidal effect of daptomycin is generally associated with an AUC/MIC ≥200 [Intensive Care Med. 2020;46:1127–1153. Expert Rev Clin Pharmacol. 2016;9:961–979. Ther Drug Monit. 2018;40:589–595.], while a C_max_/MIC ratio between 12 and 94 is considered optimal for achieving bacteriostatic effects [Expert Rev Clin Pharmacol. 2016;9:961–979.] and minimizing bacterial resistance [Infect Chemother. 2013;19:732–739.]. Higher dosages of daptomycin are recommend for the treatment of MRSA bacteremia [Clin Infect Dis. 2011;52:285–292.], and ineffectiveness in some patients may be attributed to suboptimal dosing [J Antimicrob Chemother. 2018;73(9):2277–2283.]. In this study, four patients (7%) receiving an underdose of daptomycin required a high dose to treat bacteremia. Of these four patients, two were infected with MRSE, one with *Leuconostoc lactis*, and one with *Staphylococcus epidermidis*. The high rates of microbiological failure and mortality observed in patients receiving an underdose of daptomycin highlight the importance of administering approved doses as specified in the package insert” (Page 19, line 292 to Page 20, line 302).

7. Authors need to provide the different infectious agents/bacterial species affected patient specific as additional column in a table. Ideally, the doses of daptomycin may have decided based on the MIC/MBC values depending on the bacterial species. However, it seems there is a specific dose decided to treat the patients.

Response: In this study, we focused on bacteremia and cSSTI. We have shown the infectious disease other than bacteremia or cSSTI in Supplemental Table 2. Among 54 patients, 21 patients’ blood culture samples tested positive. As you pointed out, we added the information of agents/bacterial species in Supplemental Table 5 [S5 Table. Bacteria and MIC values for patients with positive blood cultures (n = 21)]. The role of MICs for daptomycin as a prognostic marker in episodes of MSSA bacteremia has not been extensively investigated. In a study by Cervera et al., which included only patients with endocarditis caused by MSSA, MICs for daptomycin were not related to increased mortality rates [Clin Infect Dis. 2014;58:1668–1675.]. Contrarily, some reports have described its effects on microorganisms such as *Staphylococcus aureus*, *Enterococcus faecium*, and *Enterococcus faecalis*. Resistance in both *Staphylococcus* spp. and *Enterococcus* spp. is thought to be mediated by adaptations in cell wall homeostasis and membrane phospholipid metabolism [J Antimicrob Chemother. 2018;73:1–11. J Antimicrob Chemother. 2003;51:639–649. Ther Drug Monit. 2020;42:349–352.]. This study included patients whose initial blood cultures were negative, and we could not get information of MBC in medical records. Further research, including information of daptomycin dose based on the MIC/MBC values depending on the bacterial species, is needed. We added this as a limitation as follows: “Seventh, we could not investigate the association between the bactericidal effect of daptomycin and MIC/minimum bactericidal concentration (MBC). Further research, including information of daptomycin dose based on the MIC/MBC values depending on the bacterial species, is needed” (Page 23, line 372–375)

Supplemental Table 5. Bacteria and MIC values for patients with positive blood cultures (n=21)

| Case | Bacteria | MIC | Type of Infection | Sex | Age, years | Dose | Clinical effectiveness |
| --- | --- | --- | --- | --- | --- | --- | --- |
| 1 | *Leuconstoc lactis* | – | Bacteremia | F | 3 | Underdose | Failed |
| 2 | MRSA | 0.25 | Bacteremia | F | 18 | Adequate dose | Cured |
| 3 | MRSA | 0.25 | cSSTI | M | 7 | Overdose | Cured |
| 4 | MRSA | 0.5 | Bacteremia | M | 18 | Overdose | Improved |
| 5 | MRSA | <1.0 | Bacteremia | M | 16 | Adequate dose | Non-evaluable |
| 6 | MRSE | 0.5 | Bacteremia | F | 0 | Adequate dose | Failed |
| 7 | MRSE | 1.0 | Bacteremia | M | 2 | Adequate dose | Failed |
| 8 | MRSE | – | Bacteremia | F | 2 | Adequate dose | Failed |
| 9 | MRSE | 0.5 | Bacteremia | F | 1 | Adequate dose | Improved |
| 10 | MSSA | <0.25 | Bacteremia | M | 1 | Adequate dose | Cured |
| 11 | *staphylococcus epidermidis* | <0.25 | Bacteremia | F | 11 | Underdose | Failed |
| 12 | *Staphylococcus epidermidis*-MRCNS | <0.5 | Bacteremia | F | 17 | Adequate dose | Improved |
| 13 | *Staphylococcus epidermidis*-MRCNS | 2.0 | Bacteremia | M | 13 | Adequate dose | Cured |
| 14 | *Staphylococcus epidermidis*-MRCNS | <0.5 | Bacteremia | M | 17 | Adequate dose | Cured |
| 15 | *Staphylococcus epidermidis*-MRCNS, *Streptococcus oralis* | <0.5 | Bacteremia | M | 17 | Adequate dose | Cured |
| 16 | *Staphylococcus haemolyticus*-MRCNS | <0.5 | Bacteremia | M | 16 | Adequate dose | Improved |
| 17 | *Staphylococcus hominis*-MRCNS | <0.5 | Bacteremia | F | 15 | Adequate dose | Cured |
| 18 | *Staphylococcus haemolyticus* | 0.5 | Bacteremia | F | 2 | Adequate dose | Non-evaluable |
| 19 | *Staphylococcus epidermidis* | 1.0 | Bacteremia | M | 17 | Adequate dose | Improved |
| 20 | *Staphylococcus epidermidis* | 0.5 | Bacteremia | M | 17 | Adequate dose | Improved |
| 21 | *Staphylococcus haemolyticus* | 0.5 | cSSTI | M | 17 | Adequate dose | Non-evaluable |

cSSTI, complicated skin and skin structure infection; F, female; M, male; MIC, minimum inhibitory concentrations; MRCNS, methicillin-resistant *coagulase-negative Staphylococci*; MRSA, methicillin-resistant *Staphylococcus aureus*; MRSE, methicillin-resistant *Staphylococcus epidermidis*; MSSA, methicillin-susceptible *Staphylococcus aureus*

8. As, this antibiotic is primarily recommended to be used as an alternative to the patients with antibiotic resistance (MRSA, MRSE etc), the results should discuss specifically the treatment outcomes of those cases.

Response: Among the 11 patients, two were infected with methicillin-resistant *coagulase-negative staphylococci*, one with MRSA, and three with methicillin-resistant *Staphylococcus epidermidis*. The remaining patients included one with *Leuconostoc lactis*, one with *coagulase-negative staphylococci*, and three with insufficient information. Daptomycin was initiated as a second-line treatment due to adverse events or insufficient therapeutic effect from vancomycin or teicoplanin. Among these, two patients experienced adverse events, but showed therapeutic effectiveness. Three patients received underdose and unfortunately died. The remaining six patients received either the adequate dose or an overdose but showed therapeutic efficacy without adverse events. It can be concluded that daptomycin is effectiveness as a second-line treatment when administered at the approved dosage.

As per your suggestion, we have added the following information:

“Among the 11 patients who received daptomycin as a second-line treatment, two were infected with methicillin-resistant *coagulase-negative staphylococci*, one with MRSA, and three with methicillin-resistant *Staphylococcus epidermidis*. The remaining patients included one with *Leuconostoc lactis*, one with *coagulase-negative staphylococci*, and three with insufficient information. Among the six patients with antibiotic-resistant infections, one was cured, two improved, two showed partial improvement, and one was non-evaluable. Five of the six patients showed disappearance of infection, while one experienced microbiological failure” (Page 12, line 236–243).

“Daptomycin was used as a second-line treatment in 11 patients due to adverse events or insufficient effectiveness from vancomycin or teicoplanin. Among these, two patients experienced adverse events but showed therapeutic effectiveness. Three patients who received an underdose unfortunately passed away, while six patients who received adequate dose or overdose demonstrated therapeutic effectiveness without adverse events. Due to the small sample size, the impact of prior treatments on daptomycin effectiveness could not be confirmed, but future studies will aim to gather more data.” (Page 20, line 305–312)

Reviewer #2: The manuscript, titled "Influence of Renal Function and Daptomycin Dose on Clinical Effectiveness and Adverse Events in Japanese Pediatric Patients: A Multicenter Retrospective Observational Study," offers valuable insights into the clinical use of daptomycin in pediatric populations, particularly in relation to dosing and renal function. The study is well-designed, with data collected over an extensive time frame and involving multiple institutions, ensuring robust and generalizable findings. The authors report high clinical success rates and provide nuanced analyses of risk factors for adverse events, emphasizing the importance of dose optimization. The manuscript is significant as it addresses a gap in pediatric pharmacology, where evidence for daptomycin use has been limited. While the study is methodologically sound and its findings are impactful, minor revisions are necessary to improve clarity in the discussion. Please elaborate on the implications of adverse event monitoring. Overall, the manuscript presents compelling data that merit publication following minor adjustments.

Response: Thank you for your comment. CPK values were measured every 4–7 days in 23 (42%) patients, every 2–3 days in 11 (20%) patients, every 10–14 days in 9 (17%) patients, only once in 3 (6%) patients, and at >14–day intervals in 1 (2%) patient, while they were not measured during daptomycin therapy in 7 (13%) patients (Fig 2). Therefore, appropriate adverse event monitoring, including a clinical symptom should be suggested. This study had a limited sample size, and the timing of adverse events varied between cases. In pediatric patients, we aim to identify patient characteristics that are more susceptible to adverse events in the future by incorporating additional information, such as AUC/MIC values, and focusing on patient backgrounds.

Other

We have added the sentence “Three cases of treatment failure involved patients who received an underdose of daptomycin for bacteremia” in Page 11, line 224–225.

We revised “adequate dose” to “overdose” in Page 12, line 233.
