# Supplementary material for: Influence of renal function and daptomycin dose on clinical effectiveness and adverse events in Japanese pediatric patients: A multicenter retrospective observational study
Source: PLoS One. 2025 Jul 17;20(7):e0327993. doi: 10.1371/journal.pone.0327993 (PMC12270112; doi:10.1371/journal.pone.0327993)
Supplement: Attachment — Submitted filename: PONE-D-24-52543_Comments - revised.docx [file pone.0327993.s009.docx]

Reviewer #3:

Thank you for giving us the opportunity to strengthen our manuscript following your valuable comments and queries. We have incorporated your feedback and hope that our revisions persuade you to accept our manuscript.

I see that the manuscript has already undergone a round of revision but some minor issues still persists according to me. Please check that the scientific names of the bacteria has been written properly like supplemental table 5, point 11. As that seems a better and classical way of representation.

Response: I appreciate your comment. We revised “methicillin-resistant *Staphylococcus epidermidis* (MRSE) (Page 12, line 237), and changed from “*Staphylococcus*

*haemolytucus*” to “*Staphylococcus haemolyticus*” (Table2), “*Staphylococcus aureus*” to “MSSA” (Table 2), “*Leuconstoc lactis*” to “*Leuconostoc lactis*” (S5 Table), and from “*Staphylococcus epidermidis*-MRCNS” to “*Staphylococcus epidermidis*, MRCNS” (S5 Table).

For the figures added to reviewer#1 and reviewer#2 comments, it is advised the percentage of patients either cured or other conditions be depicted in bar graph with X-axis showing conditions and Y-axis showing the percentage - reverse to the present one.

Response: Thank you for your comment. As your comment, depicted in bar graph with X-axis showing conditions and Y-axis showing the percentage.

Figure3 (revised version).

Authors could also add in some survival curves with and without treatment. Also illustrate how the problem statement arises, the limitations and scope in the discussion briefly.

Response: We did not include patients who did not receive daptomycin treatment or lacked information on the time of death in this study. Therefore, we plan to further investigate the impact of daptomycin therapy and other treatment options on survival outcomes.

Reviewer #4:

Thank you for giving us the opportunity to strengthen our manuscript following your valuable comments and queries. We have incorporated your feedback and hope that our revisions persuade you to accept our manuscript.

The manuscript is well written, and the authors have made necessary changes in the manuscript to justify the concerns raised by previous reviewers. Only thing that is a bit concerning to me ethically is that, how the administration of underdosage to pediatric patients was ethically approved? As per the results of this study and other studies as well, is very evident that due to the use of underdosage of daptomycin, fetal outcomes were observed for multiple pediatric patients. The authors should address issue this in their discussion section. Although, the study was done retrospectively, still the concern remains as to how the pediatric patients were treated with underdose of Daptomycin. The authors could have compared adequate and overdose only.

Response: The dosing of DAP in the pediatric population varies by age, as younger patients exhibit increased DAP clearance and therefore require higher doses to achieve target exposures (Abdel-Rahman SM,et al. Pediatr Infect Dis. J. 2008;27:330–334). Underdosing of daptomycin has been associated with unfavorable outcomes in multiple pediatric patients (Wei XC, et al. J Clin Pharmacol. 2020;60(6):768-774. Olney KB, J Clin Pharmacol. 2024;64(7):860-865.). The specific reason for the low-dose administration in this case is unclear, but it may be related to the absence of MRSA infection. In general, low-dose administration not only contributes to the development of antimicrobial resistance but may also lead to treatment failure; thus, appropriate dosing is essential. Furthermore, a previous study revealed that the pediatric package label dosing of DAP resulted in insufficient drug exposure, with the probability of target attainment for efficacy reaching only 26.3-50.1% in pediatric patients compared to the desired goal of ≥90.0%. Based on pharmacokinetic/pharmacodynamic analysis, doses higher than those currently recommended in the pediatric labeling would be necessary to achieve validated efficacy targets (Olney KB, et al. J Clin Pharmacol. 2024;64:860–865.). Therefore, further studies investigating the relationship between DAP concentration, dosage, and clinical effectiveness are warranted.

We added the sentence as follow “The specific reason for the low-dose administration in this case is unclear; however, it is potentially related to the absence of MRSA infection” (Page 20, line 300-301).

“Furthermore, a previous study revealed that the pediatric package label dosing of DAP resulted in insufficient drug exposure, with the probability of target attainment for efficacy reaching only 26.3-50.1% in pediatric patients compared to the desired goal of ≥90.0%. Based on pharmacokinetic/pharmacodynamic analysis, doses higher than those currently recommended in the pediatric labeling would be necessary to achieve validated efficacy targets (Olney KB, et al. J Clin Pharmacol. 2024;64:860–865.). Therefore, further studies are warranted to investigate the relationship between DAP concentration, dosage, and clinical effectiveness.” (Page 20, line 303-310.)
